# Long-term follow-up of patients with chronic total coronary artery occlusion previously randomized to treatment with optimal drug therapy or percutaneous revascularization of chronic total occlusion (COMET-CTO)

**DOI:** 10.3389/fcvm.2022.1014664

**Published:** 2023-01-09

**Authors:** Stefan A. Juricic, Sinisa M. Stojkovic, Alfredo R. Galassi, Goran R. Stankovic, Dejan N. Orlic, Vladan D. Vukcevic, Dejan G. Milasinovic, Srdjan B. Aleksandric, Miloje V. Tomasevic, Milan R. Dobric, Milan A. Nedeljkovic, Branko D. Beleslin, Miodrag P. Dikic, Marko D. Banovic, Miodrag C. Ostojic, Milorad B. Tesic

**Affiliations:** ^1^Clinic for Cardiology, University Clinical Center of Serbia, Belgrade, Serbia; ^2^School of Medicine, University of Belgrade, Belgrade, Serbia; ^3^Department of Health Promotion, Mother and Child Care, Internal Medicine and Medical Specialties (ProMISE), University of Palermo, Palermo, Italy; ^4^Royal Brompton & Harefield NHS Foundation Trust, London, United Kingdom; ^5^Serbian Academy of Sciences and Arts, Belgrade, Serbia; ^6^Department of Internal Medicine, Faculty of Medical Sciences, University of Kragujevac, Kragujevac, Serbia; ^7^Dedinje Cardiovascular Institute, Belgrade, Serbia

**Keywords:** percutaneous coronary intervention, chronic total occlusion, optimal medical therapy, outcomes, long-term follow-up

## Abstract

**Background:**

The COMET-CTO trial was a randomized prospective study that assessed long-term follow-up in patients with chronic total occlusion (CTO) in coronary arteries treated with percutaneous coronary intervention (PCI) or with optimal medical therapy (OMT). During the 9-month follow-up, the incidence of major adverse cardiac events (MACE) did not differ between the two groups; no death or myocardial infarction (MI) was observed. There was a significant difference in quality of life (QoL), assessed by the Seattle Angina Questionnaire (SAQ), in favor of the PCI group. Here we report long-term follow-up results (56 ± 12 months).

**Methods:**

Between October 2015 and May 2017, a total of 100 patients with CTO were randomized into two groups of 50 patients: PCI CTO or OMT group. The primary endpoint of the current study was the incidence of MACE defined as cardiac death, MI, and revascularization [PCI or coronary artery bypass graft (CABG)]. As the secondary exploratory outcome, we analyzed all the cause-mortality rate.

**Results:**

Out of 100 randomized patients, 92 were available for long-term follow-up (44 in the PCI group and 48 in the OMT group). The incidence of MACE did not differ significantly between the two groups (*p* = 0.363). Individual components of MACE were distributed, respectively: cardiac death (OMT vs. PCI group, 6 vs. 3, *p* = 0.489), MI (OMT vs. PCI group, 1 vs. 0, *p* = 1), and revascularization (PCI: OMT vs. PCI group, 2 vs. 2, *p* = 1; CABG: OMT vs. PCI group, 1 vs. 1, *p* = 1). There was no significant difference between the two groups regarding the individual component of MACE. Six patients died from non-cardiac causes [five deaths were reported in the OMT group and one death in the PCI group (*p* = 0.206)]. Kaplan-Meier survival curves for MACE did not differ significantly between the study groups (log-rank 0.804, *p* = 0.370). Regarding the secondary exploratory outcome, a total of 15 patients died at 56 ± 12 months (11 in the OMT and 4 in the PCI group) (*p* = 0.093). The Kaplan-Meier survival curves for all-cause mortality rates did not differ significantly between the two groups (log rank 3.404, *p* = 0.065). There were no statistically significant differences between OMT and PCI groups in all five SAQ domains. There was a significant improvement in three SAQ domains in the PCI group: PL (*p* < 0.001), AF (*p* = 0.007), and QoL (*p* = 0.001).

**Conclusion:**

After 56 ± 12 months of follow-up, the incidence of MACE, as well as QoL measured by SAQ, did not differ significantly between the PCI and OMT groups.

## Introduction

Recanalization of chronic total occlusions (CTO) of the coronary arteries is certainly one of the most technically demanding procedures in interventional cardiology. Furthermore, between 15 and 20% of patients who have an indication for coronary angiography have a CTO of at least one blood vessel (1–3). However, a significantly lower percentage of these patients are candidates for percutaneous coronary intervention (4, 5). Over the years observational studies have proven that successful recanalization of CTO is associated with improved patient symptoms, quality of life, and long-term survival compared with failed recanalization (6–8). According to the observational, non-randomized data, under elective circumstances, long-term benefits of successful percutaneous coronary intervention (PCI) of CTO have been suggested: improved survival, reduced need for coronary artery bypass grafting (CABG), and a better survival of future myocardial infarctions (MI) related to non-CTO arteries (9, 10). Several randomized studies also assessed the long-term treatment outcomes of patients with CTO; however, their initial results were slightly controversial including major adverse cardiovascular events (MACE) (11–13).

COMET–CTO was one of the first randomized studies that assessed the quality of life (QoL) in patients with CTO allocated to PCI of CTO or to optimal medical therapy (OMT). The primary endpoint of the study was the QoL assessed by the Seattle Angina Questionnaire (SAQ). The results showed that there was a significant improvement in QoL in patients with one CTO on the main coronary blood vessel in patients treated with percutaneous recanalization of CTO when compared to patients randomized to OMT only (14). However, during the 9-month follow-up, the incidence of MACE was very low, with no cardiac death or MI. Therefore, the aim of this study was to determine the long-term treatment outcome (>4 years) of patients with chronic total coronary artery occlusion previously randomized to PCI of the CTO plus OMT or just to OMT. This would be one of the first research papers on this topic in elective patients.

## Materials and methods

The study design, methods (including the sample size calculation), and results were described previously (14). The study was registered on ClinicalTrials.gov (NCT02964975). Shortly, the study was designed as an open-label, randomized, prospective study which included patients with a single chronically occluded epicardial coronary artery. Between October 2015 and May 2017, 100 patients were randomized into two groups (50 pts in each group) after signing the informed consent. They participated in the study and completed the SAQ at baseline and after the 9-month follow-up. The first group was randomized to PCI of CTO with OMT and the second group of patients was randomized to OMT only.

During the long-term follow-up, patients were contacted to assess their clinical status and their adverse events. All events were recorded in the appropriate database. For the patients we were unable to reach, information was obtained from their cardiologists, general practitioners, or hospital records. The mean follow-up time was 56 ± 12 months. The primary clinical composite endpoint was the incidence of MACE, defined as cardiovascular death, nonfatal MI, and coronary revascularization (PCI or CABG). For the secondary exploratory outcomes, we analyzed the all cause-mortality rate (15). Cardiac death was defined according to the Academic Research Consortium (ARC) criteria. MI was defined with symptoms of cardiac ischemia and a troponin level of at least one value above the 99th percentile upper reference limit according to the recent guidelines (16).

### Statistical analysis

All the data were analyzed with the statistical program SPSS version 21 (SPSS, Chicago, IL). Continuous variables are presented as the mean ± SD. Categorical variables are presented as frequencies and proportions. Differences in continuous variables were assessed with Student’s *t*-test or the Mann–Whitney *U* test, following the Kolmogorov–Smirnov test. Differences in categorical variables were compared using the chi-squared test when appropriate (expected frequencies > 5; otherwise, Fisher’s exact test was used). Changes in all five components of the SAQ were tested using repeated-measures analysis of variance for each factor. SAQ domains at the baseline and after the FUP were entered as within-subject variables, whereas the study group was entered as the between-subject variable.

## Results

### Nine-month results

In terms of baseline characteristics, the compared groups were similar (14). Patient and lesion characteristics did not differ significantly between the groups at baseline (Table 1). The mean follow-up was 275 ± 88 days. In the PCI CTO group, 94% of patients had a successful recanalization of the occlusion. The mean number of implanted drug-eluting stents per lesion was 1.78 ± 1.02, while the mean stent diameter was 2.98 ± 0.35 mm. The mean length of implanted stents was 46.84 ± 26.80 mm. This study observed a significant improvement in QoL (in all five domains of the SAQ) in patients treated with PCI of CTO plus OMT when compared with patients treated with OMT only.

**TABLE 1:** Baseline patient and lesion characteristics.

Variable	OMT *n* = 50	PCI *n* = 50	*P* OMT vs. PCI	Total *n* = 100
Age (years)	63 ± 5	61 ± 7	0.107	62 ± 6
Follow-up (days)	267 ± 93	284 ± 84	0.354	275 ± 88
BMI	27.41 ± 3.46	28.40 ± 3.86	0.176	27.91 ± 3.68
Baseline creatinine (mmol/L)	84.44 ± 17.46	81.64 ± 15.27	0.395	83.04 ± 16.38
LVEF	51.34 ± 11.28	54.90 ± 9.420	0.090	53.12 ± 10.49
Male	44 (88)	38 (76)	0.118	82 (82)
Family history of CAD	23 (46)	24 (48)	0.841	47 (4)
Hypertension	43 (86)	43 (86)	1.0	86 (86)
Hypercholesterolemia	35 (70)	36 (72)	0.826	71 (71)
NIDDM	12 (24)	10 (20)	0.664	22 (22)
IDDM	6 (12)	4 (8)	0.370	10 (10)
Non-smoker	13 (26)	20 (40)	0.149	30 (30)
Ex-smoker	23 (46)	14 (28)	0.147	37 (37)
Current smoker	14 (28)	16 (32)	0.531	33 (33)
PAD	2 (4)	2 (4)	1.0	4 (4)
Previous stroke	4 (8)	1 (2)	0.169	5 (5)
Previous MI	35 (70)	29 (58)	0.211	64 (64)
In-stent CTO	3 (6)	5 (10)	0.461	8 (8)
Angina			0.769	
CCS I	11 (22)	12 (24)		23 (23)
CCS II	26 (52)	21 (42)		47 (47)
CCS III	11 (22)	14 (28)		25 (25)
CCS IV	2 (4)	3 (6)		5 (5)
Reversible ischemia	29 (58)	26 (52)	0.814	55 (55)
demonstrated	44 (88)	47 (94)	0.295	91 (91)
Presence of viability				
CTO artery			0.060	
LAD	5 (10)	12 (24)		17 (17)
Cx	6 (12)	10 (20)		16 (16)
RCA	39 (78)	28 (56)		67 (67)
Visual reference VD	3.03 ± 0.38	2.90 ± 0.30	0.60	2.96 ± 0.34
Visual length of occlusion	20.06 ± 6.40	20.46 ± 11.47	0.830	20.26 ± 9.24
Calcification			0.585	
Mild	28 (56)	33 (66)		61 (61)
Moderate	16 (32)	12 (24)		28 (28)
Severe	6 (12)	5 (10)		11 (11)
Proximal cap tapered	26 (52)	32 (64)	0.224	58 (58)
Moderate/severe tortuosity	2 (4)	4 (8)	0.513	6 (12)
“Interventional” collateral present	26 (52)	22 (44)	0.423	48 (48)
J-CTO score	1.72 ± 1.09	1.48 ± 1.27	0.219	1.60 ± 1.18
Syntax score I	9.87 ± 3.41	10.79 ± 4.89	0.822	10.33 ± 4.22
EuroSCORE II	0.87 ± 0.34	0.80 ± 0.31	0.133	0.84 ± 0.32

Data are expressed as the mean ± SD or as the number (percentage). BMI, body mass index; LVEF, left ventricular ejection fraction; NIDDM, non-insulin-dependent diabetes mellitus; IDDM, insulin-dependent diabetes mellitus; PAD, peripheral artery disease; MI, myocardial infarction; CTO, chronic total occlusion; LAD, left anterior descending; Cx, circumflex; RCA, right coronary artery; VD, vessel diameter. Presence of viability: LV normokinesis or segmental LV hypokinesis or documented viability in CTO territory.

### Long-term outcomes

Out of 100 randomized patients, 92 were available for long-term follow-up (44 in PCI and 48 in OMT group). During the long-term follow-up, a total of 15 patients died (11 in the OMT group and 4 in the PCI group) (*p* = 0.093) (Table 2).

**TABLE 2:** Clinical outcomes: long-term follow-up.

Long-term follow-up (mean 56 ± 12 months)			
	OMT	PCI	*p*
Cardiac death	6	3	0.489
MI	1	0	1
PCI–TVR	2	2	1
CABG	1	1	1
All-cause mortality	11	4	0.093

The data is numerical. PCI, percutaneous coronary intervention; OMT, optimal medical therapy; MI, myocardial infarction; CABG, coronary artery bypass graft; TVR, target vessel revascularization.

The incidence of MACE did not differ significantly between the two groups (*p* = 0.363). There was no statistically significant difference between the two groups regarding cardiac death, MI, or revascularization (Table 2). From the non-cardiac causes of death in the OMT group, there were five deaths, and in the PCI group, there was one death (*p* = 0.206). The causes of death along with some of the baseline characteristics are shown in Table 3.

**TABLE 3:** Deceased patients and their characteristics.

	CTO location	LVEF at baseline (%)	Randomization group	CTO-PCI successful	Time to death (months)	Cause of death
1.	LAD	55	OMT	/	72	CV
2.	RCA	50	OMT	/	61	COVID
3.	LAD	43	PCI	Yes	44	CV
4.	Cx	53	OMT	/	37	Traffic acc.
5.	LAD	57	OMT	/	30	CV
6.	RCA	55	OMT	/	51	Malignancy
7.	RCA	40	OMT	/	15	CV
8.	RCA	45	PCI	Yes	60	CV
9.	RCA	60	OMT	/	9	CV
10.	RCA	55	OMT	/	54	Dementia
11.	RCA	40	OMT	/	10	CV
12.	RCA	49	PCI	Yes	34	Malignancy
13.	LAD	50	PCI	Yes	24	CV
14.	Cx	30	OMT	/	37	CV
15.	RCA	55	OMT	/	45	COVID

Data are numerical. LAD, left anterior descending artery; Cx, left circumflex artery; RCA, right coronary artery; PCI, percutaneous coronary intervention; OMT, optimal medical therapy; LVEF, left ventricular function; CTO, chronic total occlusion; CV, cardiovascular; Acc, accident.

**FIGURE 1:**
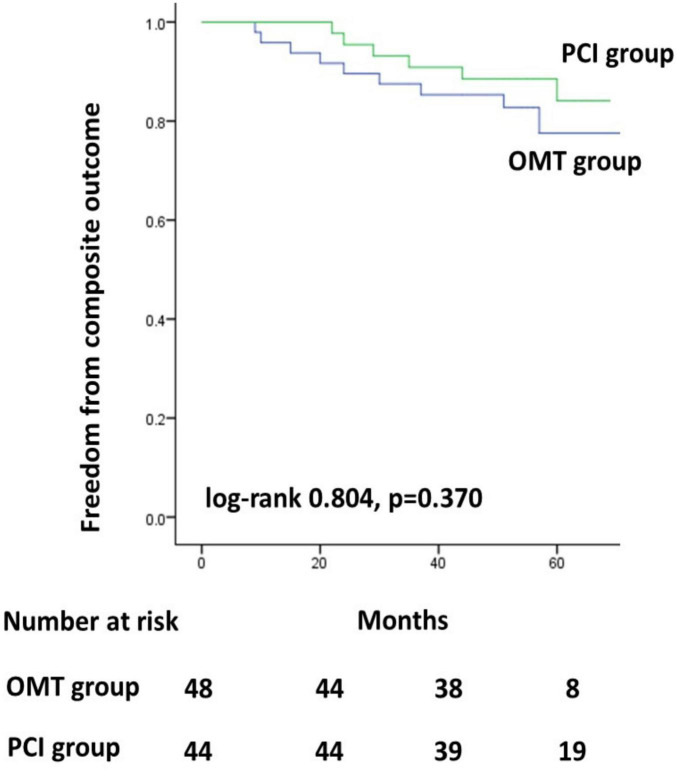
Kaplan–Meier survival curves for the MACE. PCI, percutaneous coronary intervention; OMT, optimal medical therapy.

**FIGURE 2:**
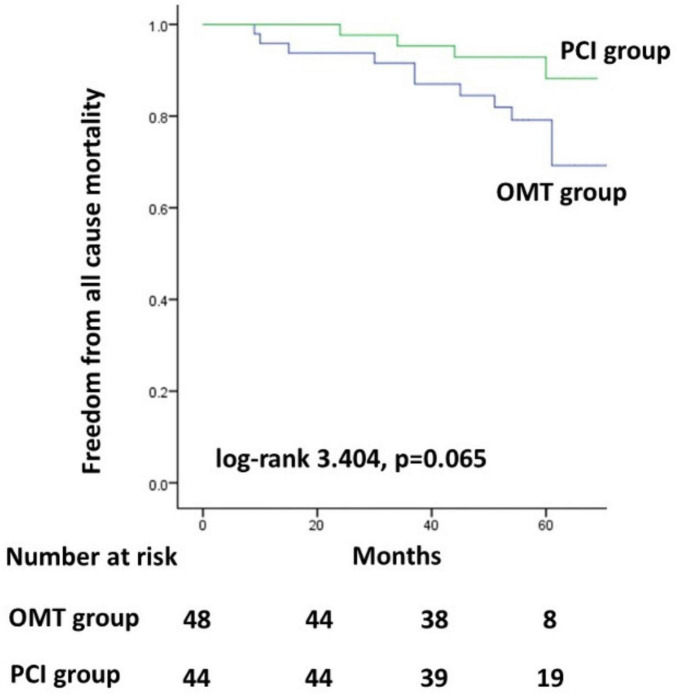
Kaplan–Meier survival curves for all-cause mortality. PCI, percutaneous coronary intervention; OMT, optimal medical therapy.

The Kaplan–Meier survival curves for the MACE did not differ significantly between the two groups (Figure 1, *p* = 0.370). In addition, the Kaplan–Meier survival curves for all-cause mortality also did not differ significantly between the two groups (Figure 2, *p* = 0.065).

After the initial FUP where we had a total of three events (one CABG in the PCI group and two cross-overs in the OMT group due to anginal complaints), during the long-term FUP, there was no statistically significant difference in revascularization between the two groups (PCI: OMT vs. PCI group, 2 vs. 2, *p* = 1; CABG: OMT vs. PCI group, 1 vs. 1, *p* = 1).

**FIGURE 3:**
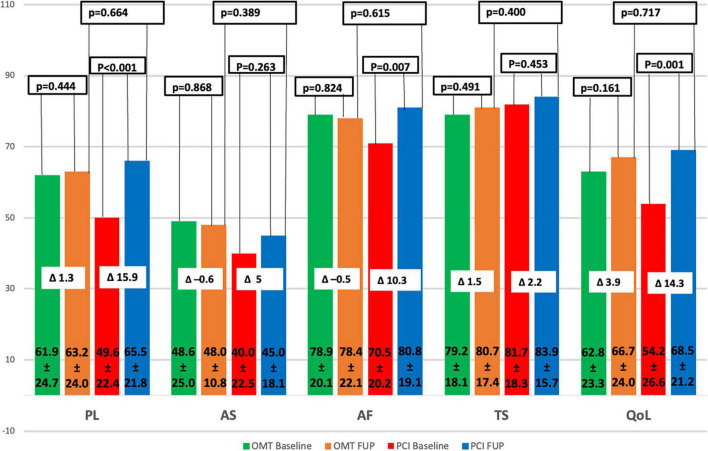
SAQ subscale changes. QoL, quality of life; PL, physical limitation; AS, angina stability; AF, angina frequency; TS, treatment satisfaction; PCI, percutaneous coronary intervention; OMT, optimal medical therapy; FUP, follow-up. Δ: difference between f-up and baseline mean values.

A total of 40 patients were analyzed for the SAQ in the PCI group and 37 patients in the OMT group. The scores for the five angina symptom domains at the FUP and their changes from the pre-procedural scores are reported in Figure 3. Baseline scores for a total of 77 patients were not different between the two groups, except for PL (PCI group 49.6 ± 22.4 vs. OMT group 61.9 ± 24.7, *p* = 0.024) (Table 4). During the FUP, there were no statistically significant differences between groups in all five SAQ domains (Figure 3). There was a significant improvement in three SAQ domains in the PCI group: PL (*p* < 0.001), AF (*p* = 0.007), and I-QoL (*p* = 0.001) (Figure 3).

**TABLE 4:** Seattle angina questionnaire health status at baseline.

	OMT *n* = 37	PCI *n* = 40	*P* *OMT vs. PCI*
**Baseline**			
PL	61.9 ± 24.7	49.6 ± 22.4	0.024
AS	48.6 ± 25.0	40.0 ± 22.5	0.114
AF	78.9 ± 20.1	70.5 ± 20.2	0.071
TS	79.2 ± 18.1	81.7 ± 18.3	0.550
QoL	62.8 ± 23.3	54.2 ± 26.6	0.133

Data are expressed as the mean ± SD. PL, physical limitation; AS, angina stability; AF, angina frequency; TS, treatment satisfaction; QoL, quality of life; OMT, optimal medical therapy; PCI, percutaneous coronary intervention.

## Discussion

To the best of our knowledge, COMET-CTO is a randomized study in elective CTO patients with the longest follow-up. The study has demonstrated that there was a trend to significantly lower all-cause mortality in the PCI group (*p* = 0.065). MACE and all the individual components of MACE did not differ significantly during the average 4.7 years of follow-up.

This study showed that QoL improved significantly in the PCI group of patients compared to the patients assigned to OMT alone. During the short-term follow-up, we observed no deaths, MIs, stent thrombosis, or strokes. However, we observed low rates of revascularization in both groups of patients. The present study extended the previous clinical outcomes from the COMET-CTO trial.

The EURO-CTO trial included 407 patients with CTO (randomization 2:1—OMT with CTO PCI or OMT, respectively) (11). In the group of patients who underwent PCI CTO, they had a significant improvement in the frequency of angina and QoL (*p* = 0.003 and *p* = 0.007), while in our study, we had a statistically significant improvement in all five domains of the SAQ. Also, the MACE rate was very similar in the EURO CTO and COMET-CTO trial during the initial follow-up.

EXPLORE (13) and DECISION-CTO (12) are two randomized studies published on this topic that did not confirm a significant reduction in MACE in the revascularized CTO group compared to the OMT group. Several things need to be addressed in both studies. Out of the 1,284 patients originally planned, only 65% (834) of the patients were included in the DECISION-CTO study. There were difficulties observed in recruiting patients which led to the main reason for the premature termination of the study. Furthermore, the significant cross-over between the two study groups (approximately one in five patients) was probably caused by the fact that PCI of non-CTO lesion was left on operator discretion before PCI of CTO. Hence, the majority of PCI of non-CTO lesions were performed after the PCI of CTO (77% of patients had multi-vessel disease).

However, overall mortality in the PCI group was lower than in the OMT group (3.0 vs. 4.4% at the 3-year follow-up and 4.5 vs. 7.9% at the 5-year follow-up). These figures did not reach statistical significance which is similar to our results.

Furthermore, in the DECISION-CTO trial, the number of patients at risk was significantly lower at the 5-year than at the 3-year follow-up (12). This could mean that the follow-up was incomplete or that a large number of patients were lost during the follow-up period. Otherwise, it is very possible that the difference would have been significant. Precisely, due to all the above-mentioned facts, the opinion of many experts in the field of CTO is that the DECISION-CTO study should not be considered as definitely negative for the primary endpoint.

In the EXPLORE trial, the MACE (composite of cardiac death, myocardial infarction, and CABG) rate did not differ significantly between the groups (13.5 vs. 12.3%, *p* = 0.93), during the median follow-up of 3.9 years. This finding is consistent with our results. An increasing trend without statistical significance was observed in the overall mortality of the CTO PCI group (12.9 vs. 6.9%, *P* = 0.11). These results should be interpreted with caution due to the small absolute number of events. Also, we cannot rule out a possibly harmful effect of PCI CTO within 7 days of an acute myocardial infarction.

Two large prospective registers, Canadian (17) and Korean (18) registers, were published in the recent years which evaluated CTO revascularization using a long-term follow-up. In the Canadian Multicenter Chronic Total Occlusion Registry 1,624 patients were assigned either to the CTO revascularized group or to the group where no revascularization of the CTO artery was performed. In the group where CTO was revascularized, there was a significantly lower mortality rate at 10 years (22.7 vs. 36.6%), lower revascularization rates (14.0 vs. 22.8%), and a shorter acute coronary syndrome hospitalizations (10.0 vs. 16.6%).

The Korean registry is a single center register with patients with CTO classified into two groups, PCI CTO (*n* = 883) or OMT group (*n* = 664), depending on the initial treatment strategy. Patients were enrolled during the period from 2003 to 2012 with typical angina or a positive functional test. At the end of the 3-year follow-up, there was no significant difference between the groups in terms of mortality due to a cardiac cause. However, between 3 and 10 years of follow-up, there was a significantly lower mortality in the revascularized group (8.3 vs. 16.6%; HR, 0.43 [95% CI, 0.27–0.71]; *P* < 0.001).

Furthermore, it is observed that the results of randomized studies and prospective registries are more similar for MACE when it comes to long-term follow-up.

In COMET CTO initial result of significant difference in QoL in favor of the PCI group after 9 months of F-up for all five SAQ domains, vanished during the next 4 years. In contrast, ISCHEMIA trial showed that QoL assessed by SAQ was significantly improved after 36 months of FUP (19). This discrepancy could be in a part explained by the fact that the number of patients available for QoL assessment decreased to 77 by time [numerically more patients died in the OMT group (11 pts) than in PCI group (4 pts)]. Bearing this in mind, it is not insignificant to emphasize that, in our trial, SAQ scores were numerically higher in four domains at the end of long-term FUP.

### Limitations

This is a prospective randomized study conducted in a single center. This study does not have statistical power to detect differences in the incidence of major adverse cardiovascular events between the examined groups (which was considered as a secondary endpoint).

## Conclusion

In patients with a single CTO treated by percutaneous coronary intervention with OMT (study group) in comparison with a group of patients only treated with OMT (control group), there was no difference observed in long-term MACE or other clinical events between the two groups. Similarly, SAQ data showed that QoL did not differ significantly between OMT and PCI groups. Further research is needed to clarify and evaluate this important area.

## Data availability statement

The raw data supporting the conclusions of this article will be made available by the authors, without undue reservation.

## Ethics statement

The studies involving human participants were reviewed and approved by the Medical Ethical Committee of the University Clinical Center of Serbia. The patients/participants provided their written informed consent to participate in this study.

## Author contributions

SJ, SS, and MBT contributed to drafting of the manuscript, analysis and interpretation of data, and discussions on the results. SJ, DM, MRD, MB, SA, and MPD collected the data. SS, SJ, AG, VV, GS, MVT, BB, MO, DO, and MN revised the final revision and final approval of the manuscript. All authors provided critical feedback and contributed to the manuscript.
